# Pancreaticoduodenectomy for Retroperitoneal Sarcomas: A Mono-Institutional Experience in China

**DOI:** 10.3389/fonc.2020.548789

**Published:** 2020-09-23

**Authors:** Cheng-Peng Li, Zhen Wang, Bo-Nan Liu, Ang Lv, Dao-Ning Liu, Jian-Hui Wu, Hui Qiu, Chun-Yi Hao

**Affiliations:** Key Laboratory of Carcinogenesis and Translational Research (Ministry of Education), Sarcoma Center, Peking University Cancer Hospital and Institute, Beijing, China

**Keywords:** retroperitoneal sarcoma, liposarcoma, pancreaticoduodenectomy, resection, survival

## Abstract

**Background:**

En bloc resection of retroperitoneal sarcoma (RPS) with adjacent organs such as pancreatic head and duodenum is challenging for surgeons. This mono-institutional study aims to evaluate the feasibility, safety, and outcome of performing pancreaticoduodenectomy (PD) during RPS resection.

**Methods:**

The clinical data of RPS patients who underwent PD at the Sarcoma Center of Peking University Cancer Hospital from January 2011 to December 2019 was collected and analyzed.

**Results:**

Twenty-seven patients out of a total of 264 surgically treated RPS underwent PD. The main pathological subtype was liposarcoma. All patients received concomitant resection of a median of three additional organs (range: 1–5), including 11 patients (40.7%) who underwent inferior vena cava resection and one patient who underwent segmental superior mesenteric-portal vein resection. Microscopic tumor infiltration to the duodenum or pancreas was observed in 81.5% of patients. Major complications occurred in 40.7% of patients; the reoperation rate was 22.2%. One patient (3.7%) died from liver abscess postoperatively. During a median follow-up of 18.9 months, 15 patients (55.6%) developed locally recurrent disease; two patients (7.4%) also had pulmonary metastases additionally. Twelve patients (44.4%) died from local relapse eventually.

**Conclusion:**

PD during RPS resection is feasible, and it may be necessary to achieve complete resection. However, considering the complexity and risk, it should be performed by an experienced surgical team. The long-term survival benefit of this procedure should be verified by further large-scale multi-institutional studies.

## Introduction

Retroperitoneal sarcomas (RPS) are rare tumors composed of numerous heterogeneous histological subtypes, with an expected incidence of less than three cases per million people each year in the United States (1). Complete resection remains the only chance of cure for RPS. Differing from sarcomas arising in the extremity and trunk, local control of RPS poses a significant challenge due to the massive size and potential involvement of adjacent organs, vessels, and other structures. The goal of surgery should be to achieve macroscopically complete resection, with a single specimen encompassing the tumor and adjacent organs, and to minimize microscopically positive margins. It is well accepted that the kidney, colon, and psoas muscle can usually be resected with relatively low morbidity. However, resections of some crucial anatomic structures such as the pancreas, duodenum, inferior vena cava (IVC), and aorta substantially are with greater risks for morbidity and death.

Pancreaticoduodenectomy (PD) itself is a high-risk operation. Clearly, RPS resection associated with PD significantly increases its complexity, difficulty, and risk. To date, there is only one multicenter report of 29 RPS patients who underwent PD from 10 sarcoma centers (2). Herein, we reported a mono-institutional experience of 27 PDs among RPS resection by a single surgical team, focusing on feasibility, safety, and outcome.

## Patients and Methods

This study was approved by the Ethics Committee of Peking University Cancer Hospital. A retrospective review of the institutional sarcoma database and patients’ clinical charts was conducted. The data of the patients who underwent PD during RPS resection was collected. The patients with subtypes other than RPS (e.g., GIST and desmoid tumor) were excluded. Written informed consent, as required by the Institutional Review Board of Peking University Cancer Hospital, was obtained from all patients. Information including previous surgical treatment for recurrent RPS patient (previous operation, histology, etc.), pathological features (tumor size, histology, margin status, microscopical infiltration to adjacent organs, etc.), operative characteristics (incision-to-suture time, estimated blood loss, blood transfusions, etc.), and postoperative outcomes (complications within the hospital stay, postoperative hospital stay, 90-day mortality, etc.) was collected and analyzed.

The postoperative clinical data was collected concerning any complication and deviation from the normal postoperative course. Postoperative pancreatic fistula (POPF), delayed gastric emptying (DGE), and postpancreatectomy hemorrhage (PPH) were separately graded in accordance with the standards published by the International Study Group of Pancreatic Surgery (ISGPS) (3–5). Bile leakage (BL) after hepaticojejunostomy or hepatic resection was graded according to the definition and grading system by the International Study Group of Liver Surgery (6). Considering grade A in these complications usually has no significant clinical impact and may lead only to a slight deviation of the clinical pathway; “major” complications were defined as grades B and C for POPF, DGE, PPH, or BL. The other postoperative complications were graded by Clavien–Dindo classification and considered “major” if Grade III or higher (7).

Surgical resection was classified as complete (R0 or R1) or incomplete (R2) because the anatomic location of RPS makes it questionable to use a reliable microscopic assessment of margins on the previous study. Pathological diagnosis was reviewed by two experienced pathologists with special expertise in sarcomas, along with microscopical tumor infiltration status to the resected organs. If needed, molecular testing (e.g., MDM2 in liposarcoma) was used to confirm the diagnosis. Patients were routinely followed up by clinical examination, laboratory tests, and CT/MRI. The primary outcomes were overall survival (OS) and disease-free survival (DFS). OS was calculated from the date of surgery to the date of death or to the last date of follow-up as the patients were alive. DFS was calculated to the date of diagnosis of locally recurrent/metastatic disease or death whichever was observed first.

Considering the new onset or worsening of diabetes mellitus after pancreatic resection is common, relevant information including preoperative and postoperative diabetic status, as well as fasting blood glucose values both at the index hospitalization and at the latest follow-up, was collected. Preoperative diabetes was defined as any patient report of diabetes or the presence of anti-hyperglycemic medication preoperatively in manner similar to the definition used in the postoperative setting.

## Statistical Analysis

Statistical analysis was performed with SPSS Statistics (IBM SPSS Statistics for Windows, Version 23.0. Armonk, NY, United States: IBM Corp.). Standard descriptive statistics were calculated for categoric data (i.e., frequency and percentage) and continuous data (i.e., median and range), as listed in the tables. Survival curves were estimated by means of the Kaplan–Meier method and were compared by the log-rank test.

## Results

### Clinicopathological and Operative Characteristics

**FIGURE 1:**
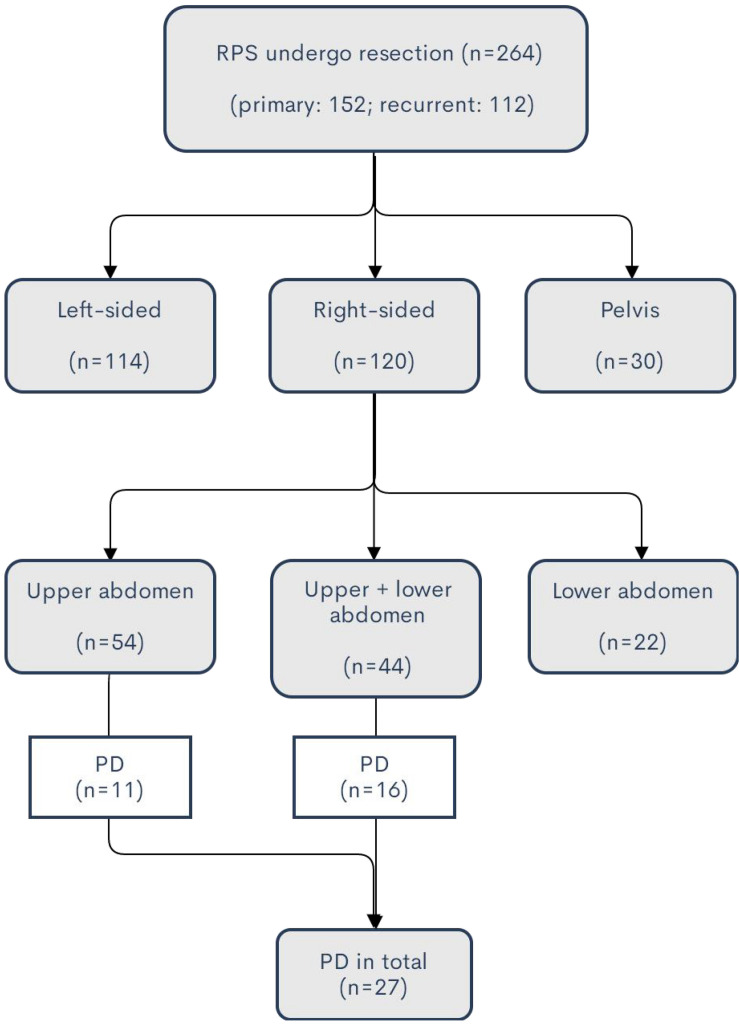
Summary of tumor locations of the patients in the study. For the huge tumor exceeding the midline or multifocal disease, the side of location is defined as the major part of the tumor location.

A total of 264 patients with RPS underwent surgical resection at Sarcoma Center of Peking University Cancer Hospital from January 2011 to December 2019. The median tumor size is 17.5 cm (range: 3.5–50 cm). Of them, 152 patients (57.6%) had primary RPS who were first seen at our institution and 112 patients (42.4%) had recurrent RPS. The median tumor size in the primary group is 16 cm (range: 3.5–45 cm) versus 19 cm (range: 4–50 cm) in the recurrent group. The tumors were predominantly located at the retroperitoneum (234 patients, 88.6%), while they were originated from the pelvis in the other 30 patients (11.4%). The details of the tumor locations were demonstrated in Figure 1.

In total, 27 patients (11 male and 16 female) who underwent PD were enrolled in the study, resulting in an overall frequency of 10.2%. The patients were grouped by primary RPS or locally recurrent RPS (LR-RPS). Fifteen patients (55.6%) had primary RPS; the other 12 patients (44.4%) of LR-RPS had an average of 1.9 (1–4) previous operations with the intention of resection elsewhere 13–157 months before admission. Of them, one previous operation was performed for 4 patients, 2 for 6 patients, 3 for 1 patient, and 4 for 1 patient. In terms of concomitant organ resection in the previous operations, five patients underwent right nephrectomy (four patients for tumor involvement, one for a massive bleeding caused by kidney injury during the resection), one patient underwent ileocecal resection, one patient underwent wedge resection of the duodenum, one patient underwent cholecystectomy for coexisting gallstone, and one patient underwent appendectomy for an unclear reason. As regards the pathology of the recurrent cases, the histologic subtypes of the latest recurrence were well-differentiated liposarcoma (WDLPS) in seven patients, dedifferentiated liposarcoma (DDLPS) in four patients, and malignant solitary fibrous tumor (MSFT) in one patient.

Clinicopathologic features including sex, age, pathological subtype, surgical margin, tumor size, numbers, and details of resected organs/vessels are presented in Table 1. Complete resection (R0/R1) was achieved in all patients. While the predominant histology was liposarcoma (24, 88.9%), 2 cases were diagnosed with undifferentiated pleomorphic sarcoma (UPS) and 1 case with MSFT. The median tumor size was 25 cm, and the maximum size reached 43 cm. Besides PD, all patients underwent contaminant resection of a median of 3 other organs; the most common organ was colon (100%) in both groups. As the right kidney was formerly resected for 5 patients with recurrent disease, 20 (90.9%) of 22 patients underwent right nephrectomy. In addition, the resection rates of IVC, diaphragm, and liver were 66.7, 40.7, and 33.3%, respectively. Segmental resection of the superior mesenteric-portal vein (SMV-PV) with an end-to-end anastomosis was performed in one patient with recurrent DDLPS. In one patient whose tumor penetrated the right diaphragm to the lower lobe of the right lung, intraoperative consultation was sought; wedge lung resection was performed by thoracic surgeons from our institute.

**TABLE 1:** Clinicopathologic characteristics of patients underwent PD during RPS resection.

	**Primary RPS (*n* = 15)**	**Recurrent RPS (*n* = 12)**	**Total (*n* = 27)**
Male:female	6:9	5:7	11:16
Age, median years (range)	52 (32–77)	58 (30–73)	57 (30–77)
Pathology			
DDLPS	8 (53.3%)	10 (83.3%)	18 (66.7%)
WDLPS	5 (33.3%)	1 (8.3%)	6 (22.2%)
UPS	2 (13.3%)	0	2 (7.4%)
MSFT	0	1 (8.3%)	1 (3.7%)
Complete resection (R0/R1), *n* (%)	15 (100%)	12 (100%)	27 (100%)
Tumor size, median cm (range)	21 (10–30)	26 (13–43)	25 (10–43)
Additional resected organs, *n* (%)			
Colon	15 (100%)	12 (100.0%)	27 (100.0%)
Right kidney	13 (86.7%)	7 (58.3%)^*a*^	20 (74.1%)
Liver			
Right hemi-hepatectomy	1 (6.7%)	3 (25.0%)	4 (14.8%)
Wedge liver resection	2 (13.3%)	3 (25.0%)	5 (18.5%)
Wedge resection of right lung	0	1 (8.3%)	1 (3.7%)
Right diaphragm	10 (60.0%)	8 (66.7%)	18 (66.7%)
No. of additional organs resected, median (range)	3 (1–4)	3 (1–5)	3 (1–5)
IVC resection	5 (33.3%)	6 (50.0%)	11 (40.7%)
Segmental resection with graft	4 (26.7%)	5 (41.7%)	9 (33.3%)
Tangential resection	1 (6.7%)	1 (8.3%)	2 (7.4%)
Segmental resection of SM-PV	0	1 (8.3%)	1 (3.7%)
Duodenum/pancreas involvement, *n* (%)			
Both	3 (20.0%)	5 (41.7%)	8 (29.6%)
Only duodenum	8 (53.3%)	6 (50.0%)	14 (66.7%)
Only pancreas	0	0	0
Neither	4 (26.7%)	1 (8.3%)	5 (18.5%)
Details of organ involvement, *n* (%)			
Duodenum	11 (73.3%)	11 (91.7%)	22 (81.5%)
Serosa	7 (46.7%)	4 (33.3%)	11 (40.7%)
Muscularis	2 (13.3%)	1 (8.3%)	3 (11.1%)
Submucosa	0	3 (25.0%)	3 (11.1%)
Mucosa	2 (13.3%)	3 (25.0%)	5 (18.5%)
Pancreas	3 (20.0%)	5 (41.7%)	8 (29.6%)
Pancreatic parenchyma	1 (6.7%)	2 (16.7%)	3 (11.1%)
Peripancreatic fatty tissue	2 (13.3%)	3 (25.0%)	5 (18.5%)

*^*a*^For five patients of LR-RPS, the right kidney was resected in the previous operations.*

The features of microscopic infiltration to the duodenum and pancreas are listed in Table 1. In 81.5% of the patients, tumor infiltration to the pancreas/duodenum was found. Taking the anatomic layer into consideration, as a hollow viscera, tumor infiltration to each layer of the duodenum was observed microscopically.

### Safety and Complications

There is no death on the operative table in this case series. Data on operative time, estimated blood loss, packed red blood cell transfusion, postoperative complications, reoperation, and postoperative hospital stay are shown in Table 2.

**TABLE 2:** Operative data and outcomes.

	**Primary RPS (*n* = 15)**	**Recurrent RPS (*n* = 12)**	**Total (*n* = 27)**
Operative time, median minutes (range)	515 (429–840)	704 (493–940)	544 (429–940)
Estimated blood loss, median ml (range) median	1500 (300–6000)	3900 (1000–7000)	2000 (300–7000)
Packed RBC transfusion, median unit (range)	4 (0–32)	14 (4–34)	10 (0–34)
**Major complications, *n* (%)**			
POPF (Grades B–C)	4 (26.7%)	4 (33.3%)	8 (29.6%)
Grade B	3 (20.0%)	2 (16.7%)	5 (18.5)
Grade C	1 (6.7%)	2 (16.7%)	3 (11.1%)
BL (Grades B–C)	2 (13.3%)	1 (8.3%)	3 (11.1%)
Grade B	1 (6.7%)	0	1 (3.7%)
Grade C	1 (6.7%)	1 (8.3%)	2 (7.4%)
DGE (Grades B–C)	2 (13.3%)	3 (25.0%)	5 (18.5%)
PPH (Grades B–C)	0	1 (8.3%)	1 (3.7%)
Anastomotic leakage following colectomy	0	1 (8.3%)	1 (3.7%)
Liver abscess	1 (6.7%)	0	1 (3.7%)
Wound dehiscence	0	1 (8.3%)	1 (3.7%)
Patients with major complications, *n* (%)	5 (30%)	6 (50%)	11 (40.7%)
Reoperation, *n* (%)	2 (13.3%)	4 (33.3%)	6 (22.2%)
Sepsis following POPF	1 (8.3%)	1 (8.3%)	2 (7.4%)
Sepsis following BL	1 (8.3%)	1 (8.3%)	2 (7.4%)
GDA stump bleeding following POPF	0	1 (8.3%)	1 (3.7%)
Anastomotic leakage following colectomy	0	1 (8.3%)	1 (3.7%)
Postoperative hospital stay, median day (range) median	23 (14–64)	30 (13–56)	26 (13–64)
Death within 90 days of surgery, *n* (%)	1^*a*^	0	1 (3.7%)
Median OS, month (95% CI)	43.3 (20.4–66.2)	19.1 (15.2–23.0)	32.6 (23.2–42.0)
Median DFS, month (95% CI)	33.1 (26.2–40.0)	10.2 (8.2–12.2)	16.3 (4.8–27.8)

*^*a*^The patient died from MODS followed by liver abscess 37 days after operation.*

The median operative time of the primary cases was 515 min; that of the recurrent ones was 704 min. The median estimated blood loss in the primary group (1500 ml) was less than that in the recurrent group (3900 ml).

Major postoperative complications (Clavien–Dindo Classification III–V) occurred in 11 patients (40.7%) with a greater incidence in the recurrent group. One patient (4.3%) died of liver abscess followed by multiple-organ dysfunction syndrome (MODS) on postoperative day (POD) 37. Six patients had re-laparotomy on POD 7–35, 4 patients (2 with POPF and 2 with BL) for sepsis followed by abdominal abscesses, and one patient for bleeding of gastroduodenal artery (GDA) stump caused by POPF on POD 12, and one patient was diagnosed with leakage of ileocolic anastomosis and received emergent ileostomy on POD 7. Wound dehiscence followed by surgical incision infection was developed in the patient with GDA stump bleeding after the reoperation; it was successfully closed by application of the negative-pressure vacuum-assisted closure (VAC) device. One patient was diagnosed with leakage of ileocolic anastomosis and received emergent ileostomy on POD 7. The other patients who suffered significant POPF, BL, or DGE were eventually successfully treated during their hospital stays. None of the patients with nephrectomy developed renal failure after the surgery. All patients who underwent IVC graft received postoperative abdominopelvic contrast CT scan before discharge; there was no thrombosis found in the prostheses. Median postoperative hospital stay was 26 days (13–64 days).

Regarding the incidence of diabetes in the study, only a 57-year-old man had diabetes before surgery. He was treated with oral metformin preoperatively but experienced worsening of the disease which needed to be controlled with insulin after PD. Excluding one patient who died during the hospital stay, 3 out of 25 patients without preoperative diabetes developed new-onset diabetes at the follow-ups. Two patients needed oral anti-hyperglycemic medication; 1 patient required insulin administration. In the cohort of patients without postoperative diabetes, the median fasting blood glucose value during the index hospitalization was 4.99 mmol/L (range: 3.94–6.83), while it was 5.35 mmol/L (range: 4.22–6.92) at the latest follow-up.

### Survival

**FIGURE 2:**
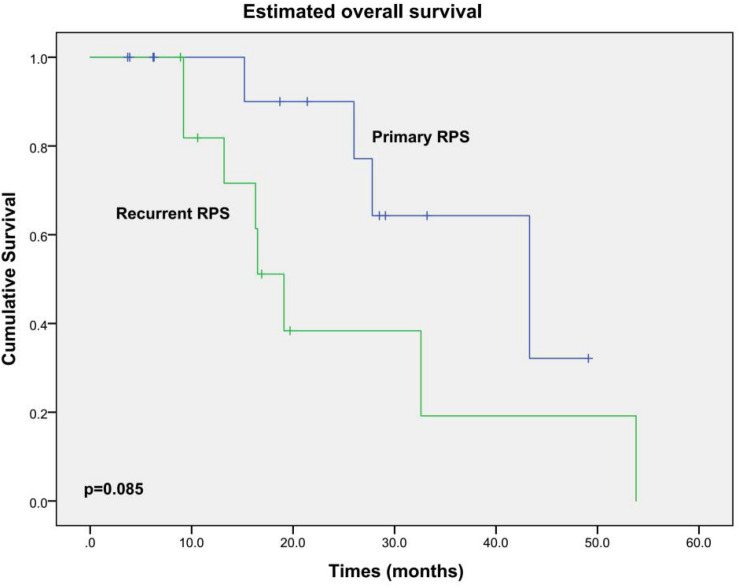
Kaplan–Meier OS estimates for the patients underwent PD during RPS resection.

**FIGURE 3:**
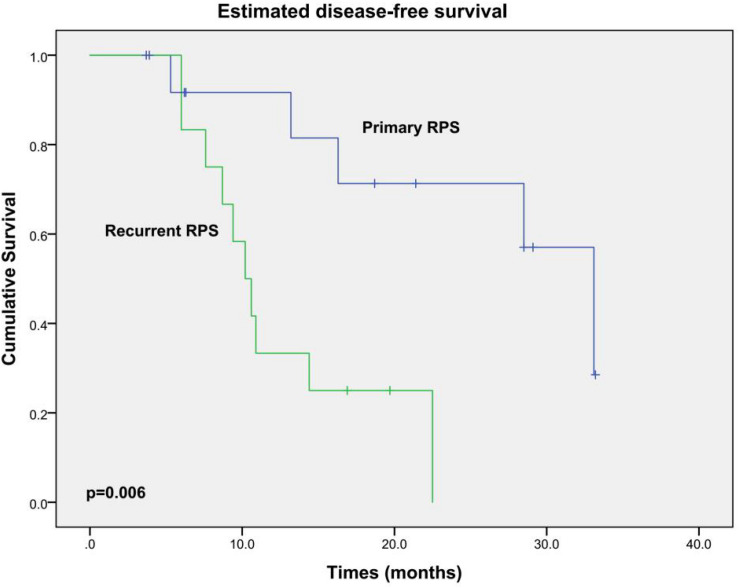
Kaplan–Meier DFS estimates for the patients underwent PD during RPS resection.

All patients did not receive any adjuvant chemotherapy or radiation therapy. Excluding one patient who died during the hospital stay, the other 26 patients were routinely followed up. During a median follow-up of 18.9 months (3.7–53.8 months), 15 patients developed locally recurrent disease; 2 of them also had pulmonary metastases additionally. Twelve patients died from local relapse eventually. OS and DFS curves are shown in Figures 2, 3, respectively. The estimated median OS (mOS) and median DFS (mDFS) of all patients were 32.6 and 16.3 months, respectively. With an mOS of 43.3 months, the primary RPS trends toward better OS in contrast with that of 19.1 months in the recurrent group, although significance was not reached likely due to the low number of patients (*p* = 0.085). The DFS of the primary group is significantly better than that of the recurrent group (mDFS: 33.1 months vs. 10.2 months, *p* = 0.006).

## Discussion

This article reported 27 PDs during RPS resection, which may be the largest mono-institutional case series in English literature at present. In the previous reports, RPS (especially retroperitoneal liposarcoma) usually tends to grow to a very large size before the presence of positive symptoms; about half of them are larger than 20 cm at diagnosis (8). In this study, the median tumor size is 25 cm. The massive tumor size and common tumor infiltration to adjacent organs and vessels created the complexity of surgical resection and difficulty in achieving wider margins. The high incidence of local recurrence after surgical resection induced an unsatisfactory long-term outcome of RPS. The Memorial Sloan Kettering Cancer Center reported that majority of patients with RPS died of advanced local tumor recurrence in the absence of systemic diseases (9). A lot of effort to improve the OS of RPS was focused on local control through adjuvant radiotherapy or surgical techniques (10). Although adjuvant radiotherapy tended to be beneficial to improve OS (11), surgery remains the mainstay of curative therapy for RPS (12). It is generally accepted that local control of RPS is mainly related to the quality of surgery. Complete resection remains one of the important predictors of outcome, which is best achieved by resecting the tumor en bloc with possibly involved contiguous organs, blood vessels, and other structures, even if exploration reveals negligible infiltration (13, 14).

In most cases of RPS, resecting the adjacent organs (e.g., kidney and colon) can be relatively safe and without severe complications (15). However, pancreatic resection often correlates with significant morbidity and mortality (16). As a high-risk procedure, PD itself is related to mortality by 2.7–2.9% in several large-scale studies (17, 18). Additional organ resections (e.g., colon) substantially increase the morbidity and mortality of PD (19, 20). Taking these factors into consideration, it may be easy to understand the frequency of PD in RPS surgery is exceedingly low even at sarcoma referral centers (2).

It is noteworthy that the frequency of PD at our institute is significantly higher than that of the previous report of TARPSWG (10.2% vs. 1.4%). We believe that it may be due to the following reasons. First, as mentioned above, PD often correlates with significant morbidity and mortality. The rarity of PD in RPS resection may not be owing to the less likelihood of tumor infiltration to the pancreas or duodenum but is probably because of surgeons’ worries about the complexities and substantially greater risks for morbidity and death of PD itself. Second, in this study, the decision to perform PD during RPS resection was based on the preoperative imaging features, intraoperative exploration, and our experience. Our previous report showed that in a group of 26 retroperitoneal liposarcoma patients who underwent pancreatectomy at our institute, 9 patients (34.6%) were found to have tumor infiltration of the pancreatic parenchyma and 6 (23.1%) showed tumor infiltration in the peripancreatic fatty tissue fat tissue (21). Such results clearly show that it may be necessary to consider performing pancreatic resection for the tumor adjacent to the pancreas, in particular, when the tumor is of great size. In such cases, performing a more conservative operation might otherwise leave microscopic disease behind. Tumor involving the duodenum is also an indication for performing PD. Although wedge/segmental resection is usually the first choice in dealing with RPS involving the duodenum, for large tumors involving the second portion of the duodenum or with simultaneous invasion of the pancreas, PD should be considered. Third, in the current study, all the operations were performed by the same surgical team; however, 29 PDs in the TARPSWG study came from 10 sarcoma centers; only 2 centers had more than 5 cases. Notably, 3 sarcoma centers with a total number of 619 RPS resections (29.9% of the entire cohort of 2068 cases) did not have any PD during the study period. As Tseng et al. discussed, the decision to perform PD may be affected by many factors including differences in institutional approaches to RPS and the individual surgeons’ familiarity with surgical management of the duodenum and head of the pancreas (2). Besides, there might be some differences in sarcoma surgeons’ concepts and training, as well as socioeconomic circumstances between centers across different countries and regions. The indications of performing PD in the management of RPS could be various at different institutes. The frequency of PD in our case series may only reflect our views and practices.

Regarding the incidence of complications, DGE and POPF are the common causes that brought about prolonged hospital stays. Sepsis followed by abdominal abscess and GDA stump bleeding is the leading cause for reoperation. Despite that the PD volume in our hospital exceeds 100 per year and almost rare postoperative deaths happened in patients who underwent standard PD, the morbidity rate in this case series is remarkably higher than that of standard PD. In consideration of the surgical complexity, proportion of previous operation history, usual malnutrition, and decreased daily activities of RPS patients, the morbidity and reoperation rate are deemed to be justified.

Comparing our results to the previous TARPSWG study of 29 patients (2), although the mortality was similar (3.7% vs. 3.4%), the major complication rate (40.7% vs. 34.4%) and reoperation rate (22.2% vs. 17.2%) are slightly higher in our study. However, considering the patients with primary RPS in our study, the major complication rate (30.0%) and reoperation rate (13.3%) are comparable to the TARPSWG data. Notably, 44.4% of patients had recurrent RPS which had an average of 1.9 previous operations and larger tumor size (median tumor size: 25.0 cm vs. 15.0 cm) in our case series; the technical difficulty in our study might be greater than in the TARPSWG study.

There is worry over the high risk of aggressive procedures such as PD in the resection of LR-RPS. Operations of recurrent diseases are more technically challenging compared to primary RPS. Handling distorted anatomic tissue plane and bowel adhesion is with a higher risk of intraoperative visceral injury, contamination, bleeding, and subsequent postoperative complications. Difficulties in the surgical treatment of recurrent RPS were directly reflected in the higher morbidity, increased operative time, and estimated intraoperative blood loss in our study. Previous data concerning morbidity and mortality of resection for LR-RPS is limited in the literature. In a study that included both primary and recurrent RPS patients who underwent resection at the University of Heidelberg, although the extent of surgery is not described, multi-visceral resection was performed in 59% of the patients, with a comparable frequency in patients with primary and recurrent tumor. In that study, there was no difference in morbidity, mortality, intraoperative blood loss, and operation time for primary versus recurrent RPS, though the morbidity was not stratified by severity (22). Generally, the treatment strategy of LR-RPS is complex and multifactorial and should be discussed in a multidisciplinary setting by a group of experienced sarcoma experts (23). As a non-standardized procedure with high mortality and morbidity, we strongly suggest that RPS patients who might have PD should be referred to high-volume surgical teams with the expertise to perform complex abdominal, sarcoma, and vascular surgeries.

Poor oncological outcome is another concern over the aggressive resection for LR-RPS (24). It is well accepted that the best chance of resection with curative intent is at the time of primary presentation. Although re-resection has been shown to extend survival and provide symptomatic relief in some patients with resectable local recurrence, there have been controversies about the appropriate treatment strategy for managing LR-RPS. Gronchi et al. reported a cohort of 167 RPS patients (82 primary and 85 locally recurrent) who underwent surgery with curative intent; the 5-year crude cumulative local recurrence rate was 36.8% in the primary group, whereas 71.5% in the locally recurrent group (25). The Heidelberg series also revealed that local tumor control after complete resection of LR-RPS was significantly inferior to primary cases; however, the 5-year overall survival rates are comparable in both groups (22). Lochan et al. reported that localized recurrence of RPS is amenable to aggressive re-resection and can lead to improved survival compared to those who did not undergo surgical resection (26). Actually, it seems that the primary cases have better survival than the recurrent ones in our study, which possibly indicates that the chance of long-term OS and DFS of recurrent RPS is limited even though complete resection was achieved. However, the size number in our study is relatively small and the survival difference between the two groups based on the statistical analysis is not solid enough. Further study in terms of a longer case series or more collaborative data should be conducted to make the results more reliable.

As an institutional approach, we commonly tend to resect the tumor en bloc with the potentially involved organs to achieve complete gross resection rather than to perform palliative resection, in the case of isolated recurrence, especially if the previous resection was an R2 resection. Re-resection for tumor residue after initial R2 resection is associated with increased morbidity and mortality; however, it may provide a similar overall survival compared to complete primary resection (27). In China, most of RPS patients had initial excision at non-specialist institutions. In our study, all the recurrent RPS cases had previous operations at outside institutes before their referrals to our center. It is probably the reason that 12 patients received 23 previous operations in total, but merely 9 organs (included 3 organs removed not as a part of en-bloc resection) were resected. Actually, as primary RPS, LR-RPS was not a homogenous group either. The decision for re-resection should be made after carefully weighing the pros and cons, based on the patient’s performance status, recurrence-free interval, and tumor biology (such as histology, grade, growth rate, response to therapy). Some histopathologic subtypes (e.g., WDLPS) would be favored for re-resection (22). In fact, WDLPS takes a major part of the previous histological subtype (58.3%) in the recurrent group of our series. Nevertheless, the chance of long-term DFS is limited after complete resection with curative intent; the decision on performing PD for patients with LR-RPS must be reached by a multidisciplinary team for the individual patient.

The present study had some limitations. First, it is a retrospective review from one single surgical team. However, as a rare subtype (RPS) within a relatively rare group (soft tissue sarcomas) in surgical practice, it is challenging for surgeons to carry out prospective randomized trials. Second, the time of follow-up in some patients is quite short, as the previous report showed RPS usually associated with late recurrence (as long as 15 years from diagnosis) in some subtypes (28). Third, the inclusion of both primary and recurrent RPS patients is a major confounding factor; we believe that further study should be focused on the patients of primary RPS, especially on the primary cases without definitely clinical or radiological evidence of tumor invasion to the pancreatic head/duodenum, to weigh the survival benefit of PD as a part of the aggressive surgical policy.

Overall, this preliminary study presented some encouraging results in the management of RPS patients with possible tumor involvement of the pancreatic head or duodenum and may be helpful for improving clinical decision-making and treatment in similar settings.

## Conclusion

Pancreaticoduodenectomy during RPS resection is feasible, and it may be necessary to achieve complete resection in cases of possible tumor involvement of the pancreatic head and duodenum; however, it should be performed by an experienced surgical team considering its great complexity and significant risk. Long-term survival benefit of PD during RPS resection, especially in the primary cases, should be verified by further large-scale multi-institutional studies.

## Data Availability Statement

The original contributions presented in the study are included in the article/supplementary material, further inquiries can be directed to the corresponding author.

## Ethics Statement

The studies involving human participants were reviewed and approved by Ethics Committee of Peking University Cancer Hospital. The patients/participants provided their written informed consent to participate in this study.

## Author Contributions

CPL, ZW, BNL, and CYH contributed to the conception and design of the study. CPL and DNL collected data and performed the statistical analysis. CPL wrote the first draft of the manuscript. JHW, HQ, and AL wrote the sections of the manuscript. All authors contributed to manuscript revision, read, and approved the submitted version.

## Conflict of Interest

The authors declare that the research was conducted in the absence of any commercial or financial relationships that could be construed as a potential conflict of interest.
